# Common mental disorders in hematopoietic stem cell transplant patients: a scoping review

**DOI:** 10.1590/0034-7167-2022-0581

**Published:** 2023-12-08

**Authors:** Ana Clara Paiva de Almeida, Valéria Dantas de Azevedo, Tássia Regine de Morais Alves, Viviane Euzébia Pereira Santos, Glauber Weder dos Santos Silva, Isabelle Campos de Azevedo

**Affiliations:** I Universidade Federal do Rio Grande do Norte. Natal, Rio Grande do Norte, Brazil; II Secretaria de Estado da Saúde Pública. Natal, Rio Grande do Norte, Brazil

**Keywords:** Hematopoietic Stem Cells Transplantation, Bone Marrow Transplantation, Mental Disorders, Mental Health, Review, Trasplante de Células Madre Hematopoyéticas, Trasplante de Médula Ósea, Trastornos Mentales, Salud Mental, Revisión, Transplante de Células-Tronco Hematopoéticas, Transplante de Medula Óssea, Transtornos Mentais, Saúde Mental, Revisão

## Abstract

**Objective:**

to map common recurrent mental disorders in patients undergoing hematopoietic stem cell transplantation.

**Methods:**

this is a scoping review carried out in January 2022 in electronic databases and repositories of dissertations and thesis. Studies that answered the research question, met the objective of the study and were available in full electronically, in any language, were included.

**Results:**

the sample consisted of 28 studies, 14 of which were published in the United States of America. The common mental disorders found were depressive, anxiety, post-traumatic stress and mood disorders. Twenty symptoms were mentioned, among the most prevalent are fatigue and sleep disorders/insomnia.

**Conclusions:**

the difficulty and importance of carrying out the differential diagnosis of these disorders were highlighted, since their symptoms can be confused with other health problems and have a strong potential to interfere with patients’ evolution.

## RESUMO

**Objetivo:**

mapear os transtornos mentais comuns recorrentes em pacientes submetidos ao transplante de células-tronco hematopoéticas.

**Métodos:**

trata-se de revisão de escopo realizada em janeiro de 2022 em bases de dados eletrônicas e repositórios de dissertações e tese. Foram incluídas publicações que respondessem à questão de pesquisa, atendessem ao objetivo do estudo e que estivessem disponíveis na íntegra em meio eletrônico, em qualquer idioma.

**Resultados:**

a amostra foi composta por 28 estudos, dos quais 14 foram publicados nos Estados Unidos da América. Os transtornos mentais comuns encontrados foram os transtornos depressivos, de ansiedade, estresse pós-traumático e de humor. Foram citados 20 sintomas, entre os mais prevalentes estão a fadiga e distúrbios do sono/insônia.

**Conclusões:**

evidenciaram-se a dificuldade e a importância de realizar o diagnóstico diferencial desses transtornos, uma vez que seus sintomas podem ser confundidos com outros problemas de saúde e têm forte potencial para interferir na evolução do paciente.

## RESUMEN

**Objetivo:**

mapear los trastornos mentales recurrentes comunes en pacientes sometidos a trasplante de células madre hematopoyéticas.

**Métodos:**

se trata de una revisión de alcance realizada en enero de 2022 en bases de datos electrónicas y repositorios de disertaciones y tesis. Se incluyeron publicaciones que respondieron a la pregunta de investigación, cumplieron con el objetivo del estudio y estaban disponibles en su totalidad en formato electrónico, en cualquier idioma.

**Resultados:**

la muestra estuvo compuesta por 28 estudios, 14 de los cuales fueron publicados en los Estados Unidos de América. Los trastornos mentales comunes encontrados fueron depresión, ansiedad, estrés postraumático y trastornos del estado de ánimo. Se mencionaron 20 síntomas, entre los más prevalentes se encuentran fatiga y trastornos del sueño/insomnio.

**Conclusiones:**

se destacó la dificultad e importancia de realizar el diagnóstico diferencial de estos trastornos, ya que sus síntomas pueden confundirse con otros problemas de salud y tienen un fuerte potencial de interferir en la evolución del paciente.

## INTRODUCTION

Hematopoietic stem cell transplantation (HSCT) consists of the infusion of hematopoietic progenitor cells, which stand out for cell dedifferentiation and self-renewal. It is a procedure that involves the elimination of patients’ hematopoietic and immune systems through chemotherapy and/or radiotherapy, with replacement by hematopoietic progenitor cells extracted from the bone marrow, by collection of peripheral blood or umbilical cord and placental blood, for the treatment of diseases onco-hematological, immunological, autoimmune, some solid tumors, among others^(1)^.

HSCT was first performed in 1957, and since then it is estimated that more than 50,000 procedures are performed annually around the world^(2)^. In recent years, the number of HSCTs performed in the United States of America (USA) has increased, reaching more than 22 thousand procedures in 2018^(3)^.

HSCT can be classified as allogeneic, with a donor with compatible human leukocyte antigen, related or not; autologous, in which patients’ own cells are applied; and syngeneic, in which the donor and recipient are identical twins^(4)^. Furthermore, HSCT is a treatment that requires long-term care from a multidisciplinary team, due to the level of complexity, aggressiveness and involvement of risks that predispose patients to a wide spectrum of complications, which need to be managed so that they do not threaten their life or affect their survival and quality of life^(5)^.

In this sense, the therapeutic process causes significant changes in patients’ daily life beyond physical complications^(1)^. Added to this is the complexity, complications and discomfort caused by the disease and therapy. Thus, there is the possibility of these abrupt changes interfering with individuals’ social and emotional domain and making them susceptible to the development of common mental disorders (CMD)^(6)^.

CMDs are a set of symptoms that do not meet criteria for the diagnosis of depression or anxiety by the Diagnostic and Statistical Manual of Mental Disorders (DSM) registered in the International Classification of Diseases 10 (ICD-10). Such disorders are characterized by non-psychotic symptoms such as irritability, insomnia, fatigue, difficulty concentrating, memory problems, feelings of worthlessness and somatic complaints, generating psychological distress and social losses for individuals^(7-8)^.

These manifestations can occur among patients undergoing HSCT as well as different forms of mental suffering even in patients with no previous psychiatric history. This fact highlights the importance of socio-emotional support before, during and after HSCT^(9-10)^.

When considering these mental comorbidities as well as the relevance of their early recognition to initiate an effective multidisciplinary approach, this research sought to answer the following guiding question: what are the most CMD in patients undergoing HSCT?

## OBJECTIVE

To map recurrent CMD in patients undergoing HSCT.

## METHODS

### Ethical aspects

As this was a study that used public domain data and did not involve human beings, there was no need for consideration by the Research Ethics Committee. However, it is worth noting that the studies selected for the final sample were properly referenced.

### Study design

This is a scoping review, developed based on Items for Systematic reviews and Meta- Analyses extension for Scoping Reviews (PRISMA- ScR)^(11)^ guidelines and guided by the JBI method Reviewer’s Manual^(12)^, with the research protocol and list of references of studies included in the final sample registered in the Open Science Framework (OSF) (https://doi.org/10.17605/OSF.IO/XMGAH).

This study aims to identify, map and synthesize the evidence and gaps in knowledge that exist around a given object of study. The elaboration process maintains methodological rigor through the following design: research question identification; search of relevant studies; study selection; data analysis and extraction; data synthesis and presentation^(11-12)^.

### Study period and location

The data collection period comprised January 2022. Study selection in databases and gray literature occurred through the Coordination for the Improvement of Higher Education Personnel (CAPES - *Coordenação de Aperfeiçoamento de Pessoal de Nível Superior*) Journal Portal, based on searches through the Federated Academic Community (CAFe - *Comunidade Acadêmica Federada*), as a way of standardizing the collection procedure in databases.

This review was developed in the following databases: PubMed; Cumulative Index to Nursing and Allied Health Literature (CINAHL); Scopus; COCHRANE; Web of Science; PsycINFO; Science Direct; Latin American and Caribbean Literature in Health Sciences (LILACS); The Education Resources Information Center (ERIC); National Library of Australia’s Trobe (Trove); Academic Archive Online (DIVA); DART- Europe E- Theses Portal; Electronic Theses Online Service (EThOS); Portuguese Open Access Scientific Repository (RCAAP); National ETD Portal; Theses Canada; CAPES Latin America Theses and Dissertations.

### Inclusion and exclusion criteria

Studies that answered the research question, met the objective of the study and were available in full electronically, through CAFe, were included. There were no language restrictions or time limits. Studies in editorial formats, letters to the editor and opinion articles were excluded. Duplicate documents were considered only once.

### Study protocol

To carry out the first stage, the mnemonic combination PCC was used (P: Population - Patients; C: Concept - CMD and C: Context - Hematopoietic Stem Cell Transplant). Therefore, the following research question was defined: What are the most recurrent CMD in patients undergoing HSCT?

In the second stage, the descriptors that represent the object of study were identified, using Medical Subject Headings (MeSH), for descriptors in English, and Health Sciences Descriptors (DeCS), for descriptors in Portuguese. The following descriptors were selected: *Pacientes*/Patients; *Transtornos Mentais*/Mental Disorders; *Transplante de Células-Tronco Hematopoéticas*/Hematopoietic Stem Cell Transplantation; *Transplante de Medula Óssea*/Bone Marrow Transplantation.

Therefore, the Boolean operators AND and OR were applied to the selected combinations. From this, it was possible to structure the search strategy as follows, in Portuguese: *Pacientes* OR (*Paciente Onco-Hematológico*) AND *Transtornos Mentais* OR (*Transtornos Mentais Comuns* OR *Saúde Mental*) AND *Transplante de Células-Tronco Hematopoéticas* OR (*Transplante de Medula Óssea*); and in English: Patients OR (Onco-Hematological Patient) AND Mental Disorders OR (Common Mental Disorders OR Mental Health) AND Hematopoietic Stem Cell Transplantation OR (Bone Marrow Transplantation).

After selecting the descriptors, a prior search for protocols was carried out in the OSF as well as in the International prospective register of systematic reviews (PROSPERO), in Cochrane Library, PubMed and also on Google Scholar with the aim of investigating the possibility of published reviews on the topic, but it was not found.

The third stage was carried out by four independent researchers, and divergences were analyzed by a fifth evaluator. The studies underwent pre-selection based on reading titles and abstracts, in order to examine consistency with the eligibility criteria listed.

In the fourth stage, texts were read in full, determining whether they actually met the inclusion and exclusion criteria.

### Analysis of results

Data collection was guided by a pre-tested form by researchers with expertise in onco-hematology and a scoping review to extract the following variables: database, language, year of publication, country of development of the study, objective of the study, methodological design, level of evidence and CMD most recurrent in patients undergoing HSCT. Thus, the data was organized and digitized into electronic spreadsheets, available in Microsoft Excel^®^ 2017. Descriptive statistics and frequencies of the data found were performed.

## RESULTS

Database searches identified 8,718,532 studies. A single filter was used to select those that were available in full and free of charge, which resulted in 6,805,343 studies, of which 1,913,189 were excluded. Subsequently, the titles and abstracts were read, in an attempt to find in these topics the theme of recurrent CMD in patients undergoing HSCT. Therefore, 6,805,258 studies did not address any of the terms in their title or abstract, and were excluded at this stage, and 85 were selected to be read in full.

**Figure 1:**
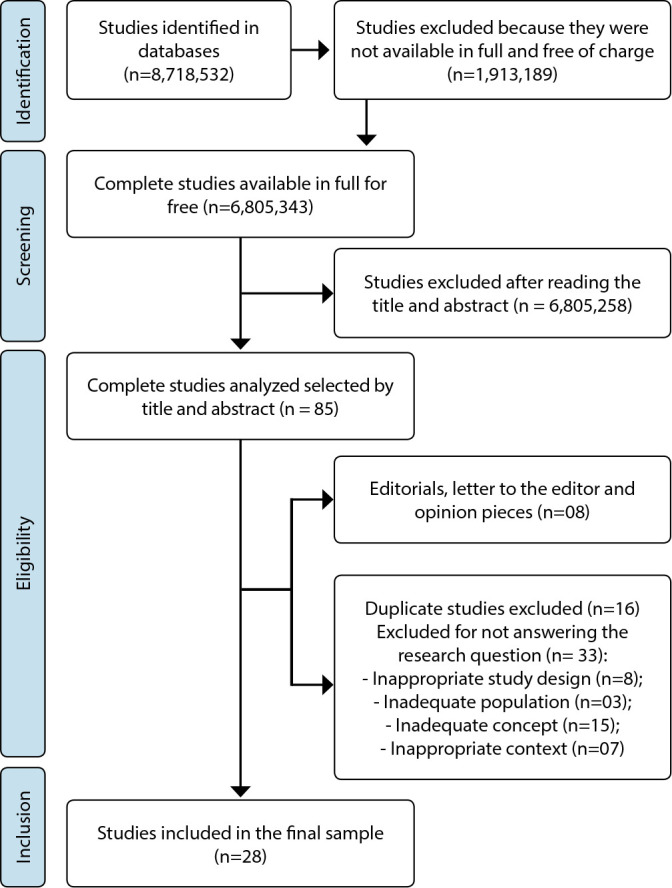
Flowchart of the study selection process for scoping review, adapted from PRISMA- ScR^(12)^, 2022

When submitted to the eligibility criteria, 33 studies did not answer the research question. Of these studies, regarding the PCC strategy, 25 were excluded, as follows: three did not correspond to the appropriate population; 15 had an inadequate concept, which focused on diagnosable mental disorders to the detriment of CMD or spoke of patients’ psychological distress, but did not specify its relationship with the HSCT phenomenon; and seven had an inadequate context, as some studies addressed CMD in onco-hematology, but not in the context of HSCT. Eight studies in the formats of editorials, letters to the editor and opinion articles were excluded. 16 studies were duplicates in the databases, which were excluded in the selection process. In total, 28 studies met the eligibility criteria and comprised the final sample of this study. A reverse search was not performed on the list of sample references. The entire study selection process is presented in the flowchart in Figure 1.

Regarding the studies found, 26 are articles and two are dissertations. Regarding language, most were published in English (26; 93%) and Portuguese (2; 7%). As for the countries in which the studies were developed, the USA, with 14 studies (50%), followed by Brazil, with four (15%), England (7%), with two, one of which is multicenter (3.5%), and the other countries such as Italy, Iran, Canada, Germany, Turkey, Poland and Australia, with one study each (3.5%), stand out.

The distribution of studies according to the year was heterogeneous and it was possible to observe the increase in studies in the second decade of the 21^st^ century in relation to the first decade. From 2002 to 2009, four studies were published (14%). Between 2010 and 2020, considerable growth was recorded, with a total of 24 studies (86%).

Four CMD diagnoses emerged: Depressive disorders cited by 19 studies (68%), followed by anxiety disorders (16; 57%), post-traumatic stress disorder (5; 18%) and mood disorders (1; 4%). 20 symptoms of CMD were highlighted in the sample, including: fatigue, sleep disorders/insomnia, changes in memory, attention, appetite, concentration and self-esteem, anguish, worry, confusion, somatic suffering, suicidal ideation, sadness, nervousness, fear, post-traumatic stress, helplessness, adjustment disorder, guilt and discouragement, the most prevalent of which were fatigue (9; 31%) and sleep disorders/insomnia (8; 28%).

Chart 1 below presents the distribution of studies according to the year, country in which the study was developed, methodological design, level of evidence and the main diagnoses of CMD and the symptoms of CMD presented by patients undergoing HSCT.

**Chart 1:** Characterization of studies regarding authorship, year, country of development, methodological design, number of patients, interventions, outcomes, diagnoses of common mental disorders, symptoms of common mental disorders presented by patients undergoing hematopoietic stem cell transplantation and level of evidence, 2022 (N=28)

ID^*^	Authors/year	Country	Design/ number of patients	Interventions	Outcomes	CMD diagnoses^†^	Symptoms of CMD ^†^	Level of evidence
A1 ^‡^	Harder H, Cornelissen JJ, Gool ARV, Duivenvoorden HJ, Eijkenboom WMH, Bent MJ (13)/2002	United States of America	Prospective cohort/40	Mini Mental State Examination, Karnofsky Performance Scale, The Groninger Intelligence Test, The National Adult Reading Test, Word Fluency Test, California Verbal Learning Test, Rey Complex Figure Test, Digit Span of the Wechsler Adult Intelligence Scale, Trail Making Test, Stroop Color Word Test, Finger Tapping Task, Reaction Time Task, Questionnaires of QOL and Mood States.	The mental status screening test revealed no abnormalities (89.8%). Mild to moderate cognitive impairment was found, and quality of life among transplant recipients in the long term (up to 10 years) still presents a wide range of persistent complaints.	-	Fatigue; sleep disorders (unspecified); memory changes; changes in attention; concentration changes	^II^I ^§^
A2 ^‡^	Fann JR, Alfano CM, Roth-Roemer S, Katon WJ, Syrjala KL(14)/2007	United States of America	Prospective cohort/90	Patients completed a battery assessing quality of life, distress and neuropsychological functioning. Delirium was assessed three times a week using the Delirium Rating Scale and the Memorial Delirium Assessment Scale from seven days before transplantation until 30 days after transplantation.	Patients with malignancy who experienced delirium during myeloablative transplantation had impaired neurocognitive abilities and persistent distress 80 days after transplantation.	Anxiety disorder; depressive disorder	Fatigue; anguish	^II^I ^§^
A3 ^‡^	Bevans MF, Mitchell SA, Marden S(15)/2008	United States of America	Prospective longitudinal/76	The occurrence of symptoms, distress and clusters was determined based on the 11 symptoms of the Symptom Distress Scale.	Allogeneic HSCT^II^ patients experience multiple symptoms and high discomfort on days 0 and 30 after transplant conditioning. On day 100, the experience is characterized by few symptoms and a low level of distress.	-	Fatigue; insomnia; concern; changes in appetite	^II^I ^§^
A4 ^‡^	Chang G, Meadows ME, Orav EJ, Antin JH(16)/2009	England	Prospective cohort/106	Application of General Social Survey prestige scales, Shipley Institute of Living Scale, Estimate a Full-Scale Intelligence Quotient Medical Outcomes Study 36-Item Short Form, Brief Profile of Mood State and neuropsychological tests.	Time and diagnosis can be important factors in the assessment of neurocognitive changes, with improvement in CMD symptoms^†^ over 18 months.	-	Memory changes; confusion	^II^I ^§^
A5 ^‡^	Wingard JR, Huang IC, Sobocinski KA, Andrykowski MA, Cella D, Rizzo JD, et al.(17)/2010	United States of America	Prospective cohort/655	Self-reported physical health was measured using the Physical Component Summary score and the Medical Outcomes Study 36-Item Short Form Health Survey.	Clinical and psychosocial factors improved among young transplant recipients, with good financial income, who had no treatment complications and fewer comorbidities over six years post-HSCT^II^.	Anxiety disorder	-	^II^I ^§^
A6 ^‡^	Basinski JR, Alfano CM, Katon WJ, Syrjala KL, Fann JR(18)/2010	Multicenter	Prospective cohort/52	Patients completed an assessment of distress, quality of life, and subjective neuropsychological functioning before receiving their first HSCT ^II^ as well as at six months and one year after transplantation.	The group that presented delirium had more fatigue and distress during treatment, and worse neuropsychological functioning at 6 months post-HSCT^II^ compared to patients who did not have an episode of delirium.	Anxiety disorder; depressive disorder	Fatigue; anguish; confusion	^II^I ^§^
A7 ^‡^	Sun CL, Francisco L, Baker KS, Weisdorf DJ, Forman SJ, Bhatia S(10)/2011	United States of America	Prospective cohort/1065	Psychological health status was assessed using the Brief Symptom Inventory-18.	The proportion of survivors reporting post-HSCT^II^ somatic distress decreased significantly over time (over 10 years). In contrast, the proportion of survivors with anxiety or depression remained unchanged during this period.	Anxiety disorder; depressive disorder	Somatic suffering	^II^I ^§^
A8 ^‡^	Tecchio C, Bonetto C, Bertani M, Cristofalo D, Lasalvia A, Nichele I, et al.(19)/2013	Italy	Prospective longitudinal/ 107	Anxiety and depression were assessed by the State-Trait Anxiety Inventory and Self-Rating Depression Scale.	One-tenth of patients suffered from anxiety and depressive symptoms on admission. Although the percentage of depressed patients more than doubled after 2 weeks of pre -HSCT^II^ isolation, that of anxious patients did not change significantly over time.	Anxiety disorder; depressive disorder	-	^II^I ^§^
A9 ^‡^	Hoodin F, Zhao L, Carey J, Levine JE, Kitko C(20)/2013	United States of America	Case-control/101	The experimental group completed the Patient Health Questionnaire briefly, focused on diagnosis to assess depressive disorders, anxiety, substance abuse, and problems in occupational or interpersonal functioning before meeting with their doctor and discussing these symptoms. The control group had access after the consultation.	The prevalence of depression in the 5-year period after HSCT ^II^ was significant, and anxiety (14%) or suicidal ideation (8%) did not differ between the 2 groups. Patients in the experimental group were significantly more likely to discuss psychological symptoms than the control group.	Anxiety disorder; mood disorders (unspecified)	Suicidal ideation	^II^I ^§^
A10 ^‡^	Crooks M, Seropian S, Bai M, Mcorkle R(21)/2014	England	Prospective cohort/ 80	Participants were given a demographic questionnaire, an emotional stress relief thermometer and a list of problems. The forms were shared with different team members and after this step, the thermometer and the list of problems were applied again at discharge, and three and six months after discharge from HSCT^II^.	Patients had an average level of emotional distress of 4.35 in the conversation about transplantation before admission; 4.42, at high; 3.54, at his three-month clinic visit; and 1.75, at six months. On average, patients’ responses revealed low to moderate levels of psychosocial distress over time, and their distress decreased at 3 and 6 months post-HSCT^II^.	-	Fatigue; sleep disorders (unspecified); sadness; nervousness; concern; changes in appetite; fear	^II^I ^§^
A11 ^‡^	Masule MS, Arbabi M, Ghaeli P, Hadjibabaie M, Torkamandi H(22)/2014	Will	Prospective longitudinal/21	Anxiety questionnaire was used and Depression Scale and Wechsler memory Scale within 72 hours after admission and one month after HSCT^II^.	It was observed that anxiety was significantly lower post-transplant compared to the pre -transplant level. However, no significant difference was found between preand post-HSCT^II^ depression.	Anxiety disorder; depressive disorder; post-traumatic stress disorder	-	IV ^¶^
A12 ^‡^	Pillay B, Lee SJ, Katona L, Burney S, Avery S(23)/2014	Australia	Retrospective cohort/122	A series of measures were completed as part of the psychological assessment of patients undergoing transplantation. They were applied the Mental Adjustment to Cancer Scale, the Brief Symptom Inventory-18 and World Health Organization Quality of Life-BREF.	In this study, 12% and 14% of the sample presented significant levels of depressive and anxious symptoms, half reported impaired physical quality of life and 40% cited poor psychological and social quality of life.	Depressive disorders; anxiety disorders	Concern; helplessness	IV ^¶^
A13 ^‡^	Artherholt SB, Hong F, Berry DL, Fann JR(24)/2014	United States of America	Prospective cohort/192	Participants were assessed pre -transplant and again 6 to 7 weeks later. Measures assessed included Symptom Distress Scale, the EORTC core quality of life questionnaire for quality of life, a single-item pain intensity question and the Patient Health Questionnaire-9 for measuring depression.	Although few patients (6%) met criteria for moderate or high depression before transplant, nearly a third (31%) met criteria for moderate or high depression when assessed six to seven weeks after transplant.	Depressive disorder	-	^II^I ^§^
A14 ^‡^	Rocha V, Kalinke LP, Felix JVC, Mantovani MF, Maftum MA, Guimarães PRB(25)/2015	Brazil	Analytical longitudinal/25	Data collection occurred through sociodemographic and clinical data questionnaires, the Quality of Life Questionnaire-C30 (version 3.0, Portuguese, Brazil) and the Functional Assessment Cancer Treatment-Bone Marrow Transplantation (version 4.0, Portuguese, Brazil).	They demonstrated a statistically significant worsening in global quality of life, functional scales, physical, social and family function, personal performance and additional concerns in the baseline, pancytopenia and prehospital discharge periods.	-	Fatigue; insomnia	^II^I ^§^
D1 ^**^	Proença SFFS(26)/2015	Brazil	Analytical longitudinal/25	Questionnaires were applied to adult cancer patients 100 days after the HSCT^II^: sociodemographic and clinical data version 2.0; EORTC core quality of life questionnaire, validated for Brazil to assess general quality of life; and Functional Assessment of Cancer Therapy Bone Marrow Transplantation version 4.0, Portuguese, validated for Brazil, specific for assessing quality of life in transplantation.	It was possible to observe that, at the end of hospitalization, patients have a quality of life with greater impairment in physical function, personal performance, symptoms (fatigue, loss of appetite, nausea and vomiting), social and family well-being, well-being functional, additional concerns, and 100 days after transplantation, patients recover baseline values, with the exception of functional well-being.	-	Fatigue; insomnia	^II^I ^§^
D2 ^**^	Marques ACB(5)/2016	Brazil	Analytical longitudinal/45	Data were collected using the following instruments: sociodemographic and clinical data; updating sociodemographic and clinical data; global quality of life; EORTC core quality of life questionnaire; and Functional Assessment of Cancer Therapy Bone Marrow Transplantation^II^ version 4.0.	The results express changes in patients’ quality of life, which translate into symptoms that go beyond physical complications, as they reveal emotional and social weaknesses present during treatment and that have disabling potential and negatively influence these patients, especially in the early stages of treatment. treatment. On the other hand, the analysis of results also showed that surviving patients consider their quality of life to be satisfactory after one year of transplantation.	-	Fatigue; insomnia	^II^I ^§^
A15 ^‡^	El- Jawahri A, Vandusen H, Traeger L, Fishbein JN, Keenan T, Gallagher ER et al.(27)/2016	United States of America	Prospective longitudinal/90	Questionnaires such as the Functional Assessment of Cancer Therapy-Bone Marrow Transplantation, Posttraumatic Stress Disorder Checklist, Hospital Anxiety and Depression Scale and the Patient Health Questionnaire were applied.	There was a decline in quality of life and an increase in depressive symptoms six months after hospitalization for HSCT ^II^	Anxiety disorder; depressive disorder	-	^II^I ^§^
A16 ^‡^	Ghazikhanian SE, Dorfman CS, Somers TJ, O’Sullivan ML, Fisher HM, Edmond SN, et al.(8)/2017	United States of America	Prospective longitudinal/138	Preand post-HSCT^II^ questionnaires were applied who assessed sleep and cognitive problems as well as commonly concomitant symptoms such as depressive symptoms, fatigue and pain.	Sleep problems are associated with and may contribute to cognitive problems in patients over the six months post-HSCT ^II^	Depressive disorders	Fatigue; sleep disorders (unspecified)	^II^I ^§^
A17 ^‡^	Kroemeke A, Kwissa Gajewska Z, Sobczyk-Kruszelnicka M(28)/2018	Poland	Cross-sectional/290	Center for Epidemiological Studies Depression Scale, Hospital Anxiety and Depression Scale, quality of life, EORTC core quality of life questionnaire, New General Self-Efficacy Scale and transplant assessment were assessed in participants.	Four latent well-being profiles were identified: good functioning (51%, greater well-being in all aspects); dysfunctional (10%, weaker functioning in all aspects); 2 profiles with moderate and high (5.6%) or low (33.4%) quality of life; and anxiety and depressive symptoms in the pre-HSCT^II^ period.	Depressive disorders; anxiety disorder	-	IV ^¶^
A18 ^‡^	Penalba V, Asvat Y, Deshields TL, Vanderlan JR, Chol N(29)/2018	United States of America	Cross-sectional/351	Questionnaires were applied to assess distress, anxiety, depression and quality of life using validated instruments, such as the Functional Assessment of Cancer Therapy-General.	The subset of patients who presented for pre -transplant psychosocial assessment subsequently utilized psychotherapy services and these reported low levels of distress, depression or anxiety.	Anxiety disorder; depressive disorder; post-traumatic stress disorder	-	IV ^¶^
A19 ^‡^	El- Jawahri A, Pidala J, Khera N, Wood WA, Arora M, Carpenter PA, et al.(30)/2018	Multicenter	Prospective cohort/482	It was examined the relationship between self-reported symptoms of depression or anxiety (measured by the Lee Symptom Scale) and patients’ quality of life (Functional Assessment of Cancer Therapy-General and the Physical Component Scale of the 36-item Short-Form Health Survey), functioning physical (measured by the human activity profile), functional status (measured by the 2-minute walk test) and overall survival.	The findings suggest that patients with self-reported symptoms of depression or anxiety present substantial impairments in their physical functioning, functional status and general quality of life in the post-HSCT^II^ period and that they developed graft-versus-host disease.	Anxiety disorder; depressive disorder	-	^II^I ^§^
A20 ^‡^	Esser P, Kuba K, Ernst J, Mehnert-Theuerkauf A(31)/2019	Germany	Cross-sectional/300	The following instruments were applied: Structured Clinical Interview, European Organization for Research and Treatment of Cancer Quality of Life Questionnaire, Depressive and Anxious Symptomatology, General Distress (Distress Thermometer), Fear of Progression, Comorbidity, Adjustment Disorder Symptomatology, Posttraumatic Stress Disorder Symptomatology, Posttraumatic Growth Inventory, Experiential Avoidance.	The prevalence of stress-related disorders and the level of distress among patients with hematological cancer in different treatment settings was verified.	Anxiety disorder; depressive disorder; post-traumatic stress disorder	Difficulty in adapting	IV ^¶^
A21 ^‡^	Nelson AM, Juckett MB, Coe CL, Costanzo ES(32)/2019	United States of America	Prospective longitudinal/332	Participants completed measures of illness perception (beliefs about the consequences and course of cancer, personal and treatment control over cancer, and understanding of cancer itself) before transplantation. Health practices (diet, physical activity and alcohol use) and mental health (depression, anxiety and psychological well-being) were assessed before transplantation and at 1, 3, 6 and 12 months after transplantation.	Mixed-effects linear regression models revealed that HSCT^II^ recipients who perceived the consequences of their cancer as more severe experienced more depression and anxiety, less well-being, and ate a healthier diet, but were less physically active during the year after transplant.	Anxiety disorder; depressive disorder	-	^II^I ^§^
A22 ^‡^	Erden S, Kuşkonmaz BB, Çetinkaya DU, Ünal F, Özsungur B(33)/2019	Turkey	Cross-sectional/30	All children were interviewed using the Kiddie Schedule for Affective Disorders and Schizophrenia, Children’s Depression Inventory, State-Trait Anxiety Inventory for Children, State-Trait Anxiety Inventory, and Rosenberg Self-Esteem Scale.	During the transplant process, children showed a higher prevalence of depression, anxiety disorder and attention deficit/hyperactivity disorder, and non-donor siblings showed a higher prevalence of depressive disorder, anxiety and attention deficit/hyperactivity disorder compared to society in general.	Anxiety disorder; depressive disorder	Changes in self-esteem	^II^I ^§^
A23 ^‡^	Azevedo IC, Ferreira Júnior MA, Flores VGT, Gonçalves EAP, Frota OP, Cardoso MP, et al.(9)/2019	Brazil	Cross-sectional/43	Medical records of 43 patients who underwent HSCT^II^ were assessed.	Among 43 patients with psychological disorders, 51.16% were female. It was observed that patients presented psychological distress from the hematological neoplasia diagnosis until the end of treatment.	Anxiety disorder; depressive disorder	-	^II^I ^§^
A24 ^‡^	Liang J, Lee SJ, Storer BE, Shaw BE, Chow EJ, Flowers ME, et al.(34)/2019	United States of America	Cross-sectional/1024	Patients completed self-report measures of sociodemographic information. Clinical variables were captured in the transplant database.	Patients or caregivers who had post-traumatic stress disorder reported significantly greater distress related to uncertainty, family tension, medical demands, finances, identity, and health burden in the post-HSCT^II^ period (7 to 10 years).	Anxiety disorder; depressive disorder; post-traumatic stress disorder	Sleep disorders (unspecified)	IV ^¶^
A25 ^‡^	Amonoo HL, Brown LA, Scheu CF, Harnedy LE, Pirl WF, El- Jawahri A, et al.(35)/2020	United States of America	Qualitative study/21	Interviews explored symptoms of psychological distress in hospital and during the first 100 days after transplant, along with the perceived impact of these symptoms on their recovery.	Of the negative emotional experiences reported, feeling trapped, fear, guilt, discouragement and helplessness were frequently expressed in the post-HSCT^II^ period.	Depressive disorder	Fear; guilt; discouragement	IV ^¶^
A26 ^‡^	Lemieux C, Ahmad I, Bambace NM, Bernard L, Cohen S, Delisle JS, et al.(36)/2020	Canada	Retrospective cohort/47	The EQ-5D instruments were used to assess mobility, self-care, usual activities, pain/discomfort and anxiety/depression, and the Functional Assessment of Cancer Therapy-Bone Marrow Transplant was used to assess physical, social/family, emotional well-being and functional and transplant-specific concerns.	The results of the EQ-5D and Functional Assessment of Cancer Therapy-Bone Marrow Transplant demonstrate that advanced age at transplantation is not a factor that impacts quality of life in post-HSCT ^II^ patients for lymphoma treatment.	Anxiety disorder; depressive disorder; post-traumatic stress disorder	-	IV ^¶^

Note:

* ID - Identification;

† CMD - Common Mental Disorder;

† A - article;

§ II I - Evidence from a cohort study, prospective longitudinal study, analytical observational study or control group study;

II HSCT - Hematopoietic Stem Cell Transplant;

¶ IV - Evidence from literature review, cross-sectional study, systematic review, narrative study or retrospective study; ^^**^^ D - Dissertation.

## DISCUSSION

Historically, health professionals have observed the physical and psychological burden that patients undergoing HSCT carry and defined it as an expected and non-modifiable phenomenon, as they face a high emotional level during periods of treatment that can result in anxiety, depression or even both^(7)^.

In this context, the present study showed the predominance of depressive disorders^(8-10,14,18-19,22-24,27-36)^ and anxiety^(9-10,14,17-20,22-23,27-33)^ as the most recurrent CMD diagnoses among HSCT patients. A variety of physical and psychological symptoms of these diagnoses, often non-specific, can be confused with common reactions to treatment and, therefore, go unnoticed^(7)^.

The USA stood out among the countries with the highest prevalence of CMD and pointed out as the main causes of prolonged hospital stay, social isolation, fear of end of life and recurrence of disease^(10,17,34-35)^. Its prevalence is related to the history of mental disorders and the female sex. The findings of this study highlighted the presence of CMD in patients aged 18 to 50 years^(5,10,13-14,17,20,31)^. From a gender perspective, the presence of CMD symptoms in the female population can be observed, mainly evidenced by an Italian^(19)^, an American^(10)^ and four Brazilian^(5,9,25-26)^ studies. Estimates vary between regions, from a low of 2.6% among men in the Western Pacific region to 7.7% among women in the Americas^(37)^.

A multicenter study carried out in Brazil showed the presence of CMD mainly in women in all cities in the country^(38)^. The female population affected by hematological neoplasms who undergo HSCT experience concerns related not only to their health problem and treatment, but to social distancing, from their family and children or even about the possibility of becoming infertile. Such feelings negatively influence their quality of life and continuity of treatment^(5)^.

HSCT generates significant psychological suffering in patients due to the threat of mortality, frequently undergoing heavy chemotherapy and shortand long-term side effects related to the treatment stages and mainly the social isolation caused by hospital admission^(35)^. Furthermore, the verification of post-traumatic stress in transplant patients is related to anguish due to uncertainty, family tension, financial problems, health burden and the use of psychotropic medications for anxiety, depressive and sleep disorders^(34)^.

HSCT can be considered a traumatic event by those who experience it, as it is an aggressive treatment, which exposes patients to invasive procedures, immunosuppressants that compromise patients’ quality of life, prolonged hospitalizations associated with social isolation, family overload and the caregiver, emotional and psychological conditions as well as fear and the threat of death. Symptoms of post-traumatic stress are commonly reported in this context, especially in the six months after HSCT, whether in autologous or allogeneic transplantation^(34,39)^.

Given these predictive factors for the emergence of CMD during HSCT, a study carried out in Florida, USA, studied the relationship between coping strategies and CMD symptoms. Coping is defined as a coping strategy that manages stress through cognitive efforts, which can be adaptive, when individuals engage with the problem in a constructive way, or maladaptive, such as running away from the problem or blaming other people. Therefore, it was evidenced that patients undergoing HSCT who have good coping strategies and family support demonstrate psychological resilience, even when they experience negative situations or CMD symptoms are detected^(40)^.

The number of individuals with CMD is increasing globally, especially in low-income countries, due to the increase in population and life expectancy^(37)^. The prevalence of CMD is high among lowand middle-income countries, especially Brazil. However, it is considered a psychiatric morbidity also experienced by developed countries, such as Europe^(38)^.

Depressive disorder is defined by a period of at least two weeks of depressed mood, almost always accompanied by disinterest or pleasure in activities that were previously considered enjoyable. A set of symptom manifestations may accompany these key characteristics of depression, such as changes in appetite, sleep disturbances, agitation or psychomotor retardation, fatigue, discouragement, feelings of excessive guilt and worthlessness, decreased concentration and suicidal ideation^(41)^.

Anxiety disorder is manifested by excessive fear, worry, anxiety, behavioral changes, fatigue, lack of concentration, irritability, sleep disorders, such as insomnia and impaired concentration. The emergence of these symptoms can be attributed to several factors, such as patients’ previous history, current illness process and treatments^(41)^.

Furthermore, CMD symptoms such as anguish and worry are natural emotional reactions to this entire process faced by patients, but which, when expressed excessively and recurrently, can represent mental suffering. In addition to these, there are also feelings of fear, helplessness and despair, which, despite occurring less frequently, are also considered manifestations of CMD^(7,15)^.

There are also countless other expressions of mental suffering caused by the transplant process. Impatience, emotional difficulties and mood changes were also found during the research as well as suicidal ideation, which can be observed in response to intense stressors, commonly accompanied by other symptoms, and can be mainly related to relapse and previous psychiatric disorder^(32,40)^.

HSCT is demarcated by three phases, pre-HSCT, Day Zero and post-HSCT, the latter being the most favorable period for the development of CMD. In autologous HSCT, the first phase corresponds to pre-HSCT, i.e., before the infusion of hematopoietic stem cells (HSC) and conditioning of the collected HSCT. Day Zero consists of the infusion of CTH. Post-HSCT is divided into immediate, considered up to 100 days after HSC grafting, and late, which corresponds to patient discharge^(42)^.

With regard to HSCT, CMD symptoms can appear at any time during treatment, especially between the first 100 days after the procedure and up to 10 years after the transplant^(13-18,20,22-23,25-27,30,32,34-36)^ and are related to an up to three times higher risk of death at this stage^(7)^.

However, the conditioning period is responsible for causing the highest levels of anxiety among patients, caused by isolation, insecurities about the transplant and physical responses, fear of recurrence as well as missing family, friends, breaking routine, among many other concerns^(7,9)^.

The occurrence of CMD, more specifically post-traumatic stress disorder, in the post-HSCT period is related to the experiences that patients experienced during hospitalizations throughout treatment. Toxicities occurring during the HSCT period and hospitalizations have significant shortand long-term repercussions that affect patients’ quality of life, physical and psychological well-being^(27)^.

Some of the CMD symptoms found are related to patients’ neurocognitive function, such as mental confusion, changes in memory, attention and concentration. This type of dysfunction is usually noticed in around 60% of patients between 22 and 82 months after HSCT, and is related to greater morbidity and mortality after the procedure. This is justified by greater difficulties in dealing with physical and psychological symptoms after transplantation and in maintaining health processes independently and autonomously^(7)^.

The persistence of these CMDs, such as anxiety disorder, followed by depressive disorder and the manifestation of symptoms such as fatigue and sleep disorders, with insomnia, are capable of strongly influencing a patient’s clinical evolution, are related to greater morbidity and mortality after HSCT and require attention from the health service. The population undergoing HSCT is susceptible to CMD and various other forms of mental suffering, it is up to the health team to be prepared to identify, treat and maintain follow-up of these patients^(11,17)^.

In this context, nursing is considered as the profession that is closest to patients during all phases of HSCT. It offers emotional support, comfort and enables the improvement of quality of life through the development of care plans aimed at patients at all stages of transplantation^(39)^.

The interest in researching this topic is emerging, as evidenced by this review, in which it was possible to see an increase in studies in the 21st century, the period that followed the first HSCT. However, it was in 2019 that there was the largest number of studies published on HSCT and CMD.

The initiative to study the mental health of oncohematology patients undergoing HSCT in 2019 is in line with the initiative focused on mental health launched by the World Health Organization, “The WHO special initiative for mental health (2019-2023): universal health coverage for mental health”, with the aim that all people can achieve the highest standard of mental health and well-being. In this way, research related to this topic can be promoted^(43)^.

The substantial burden that CMD causes in the life of patients undergoing HSCT can be reduced through multidisciplinary care that supports them in all phases of treatment. The remodeling of assistance based on promoting the mental health of groups most vulnerable to developing CMD has a positive impact on improving quality of life and coping with difficulties and traumatic situations^(41)^.

In this way, individual interventions, such as psychotherapy and groups, encourage the exchange of experience, strengthening bonds, improving mental health and CMD. Therefore, these actions must be initiated and encouraged from patients’ entry, during hospitalization and in the post-HSCT period, with the monitoring of Primary Health Care, duly trained to welcome them and offer this support^(44)^.

### Study limitations

This scoping review has as its main limitations the inclusion of only publicly available studies in full, since relevant studies may have been lost as they do not meet this criterion, in addition to the low level of evidence (III and IV) of selected studies and its methodological fragility. A reverse search was also not carried out in the list of references of the studies selected for the final sample.

Another limitation found is related to the explicit clinical focus in the studies, in which there is a greater interest in studies in the repercussions that HSCT causes physically to patients instead of the psychological aspect, in addition to the incipience of studies developed by nursing in this area.

### Contributions to nursing

The present study makes important contributions to the health area, especially to HSCT services, by highlighting particularities of the mental health of patients who are weakened in the face of a process as complex and exhausting as transplantation. It also shows how they are intrinsically linked to physical and mental health and the health care provided, which are interdependent and, therefore, demand quality attention and care.

The nursing team provides care directly to patients 24 hours a day, therefore, the scientific evidence elucidated by this review may encourage nursing praxis in this scenario, in addition to enabling professionals to identify changes in evolution to improve or worsen the condition patients’ health-illness framework, present some of the causal relationships between the experiences experienced throughout the HSCT context and the development of CMD.

Furthermore, it is suggested that new studies with strong scientific evidence (I and II) be developed in this area in order to investigate and relate the triggering factors for the development of CMD with HSCT and, in this way, plan the assistance of these patients based on mental health promotion throughout the course of treatment.

## CONCLUSIONS

The present study concluded that patients undergoing HSCT constitute a population vulnerable to developing CMD, due to therapeutic complexity, emotional fragility and traumatic experiences related to treatment. In view of this, it mapped the main most prevalent CMDs, including depressive, anxiety, post-traumatic stress and mood disorders and their associated symptoms, which occur mainly in the post-HSCT period.

It is worth noting that there has been a growing production of new knowledge on this topic and that this is a public health problem not only in Brazil, but also in low-, middleand high-income countries. Therefore, it is necessary to develop a care plan focused on the needs of each patient, ensuring comfort and confidence throughout all phases of HSCT.

Therefore, it is essential to include the assessment of the various aspects of mental health within nursing consultation and to qualify professionals to carry it out properly, given that nursing is the professional category most present throughout the care process, with opportunities for identify signs and symptoms in patients that may be related to CMD.
